# Supplementary material for: A global analysis of bird plumage patterns reveals no association between habitat and camouflage
Source: PeerJ. 2016 Nov 9;4:e2658. doi: 10.7717/peerj.2658 (PMC5111890; doi:10.7717/peerj.2658)
Supplement: Supplemental Information 1 [file peerj-04-2658-s002.docx]

Table S1. The number of occurrence of plumage patterns in the class Aves. Species numbers are given per pattern type, per body surface adult males and females, as well as juveniles in the breeding and non-breeding season.

|  |  | Mottled | | | Barred | | | Scaled | | | Spotted | | |
| --- | --- | --- | --- | --- | --- | --- | --- | --- | --- | --- | --- | --- | --- |
| Class | Season | Ventral | Dorsal | Both surfaces | Ventral | Dorsal | Both surfaces | Ventral | Dorsal | Both surfaces | Ventral | Dorsal | Both surfaces |
| Female | Non-breeding | 860 | 1081 | 1508 | 533 | 679 | 539 | 163 | 269 | 163 | 127 | 291 | 314 |
| Female | Breeding | 864 | 1085 | 1514 | 500 | 624 | 978 | 162 | 202 | 321 | 300 | 157 | 427 |
| Male | Non-breeding | 689 | 954 | 1288 | 530 | 876 | 535 | 153 | 277 | 162 | 122 | 385 | 314 |
| Male | Breeding | 674 | 936 | 1261 | 514 | 478 | 975 | 161 | 156 | 320 | 305 | 104 | 427 |
| Juveniles | N/A | 873 | 914 | 1368 | 886 | 325 | 627 | 274 | 158 | 203 | 385 | 223 | 157 |

**Phylogenetic comparative analyses for barred versus mottled plumage patterns**

We ran analyses to examine if habitat selects for plumage patterns at the global scale also comparing directly barred and mottled plumage patterns. Table S2 presents the results for the PhyLoRegs models for the global comparative analysis using barred and mottled plumage patterns only.

Table S2. Relationship between plumage pattern type (barred versus mottled) and habitat (using our *habitat coverage* measure) across the class Aves using Phylogenetic Logistic Regressions (PhyLoRegs) and Generalized Linear Models (GLMs). We present the estimate of the intersect as well as the slope and its associated p-value for both PhyLoRegs and GLMs. For PhyLoRegs, values correspond to the mean of 100 runs using randomly sampled phylogenetic trees from Jetz et al. (2012), and values in brackets correspond to the standard deviation. Positive slopes indicate that regular patterns are more associated with open habitat while irregular patterns are more associated with closed habitat, and negative slopes indicate the opposite. *a* is the phylogenetic correlation parameter calculated from the PhyLoRegs. R^2^ values were computed for the GLMs and correspond to the McFadden’s pseudo-R^2^. Season: NB = Non-breeding; BR = Breeding.

| **Biological combination** | | |  |  | **PhyLoRegs results** | | | |  | **GLMs results** | | | |
| --- | --- | --- | --- | --- | --- | --- | --- | --- | --- | --- | --- | --- | --- |
| **Sex** | **Body part** | **Season** |  | **Number of species** | **Intercept** | **Slope** | **P-value** | ***a*** |  | **Intercept** | **Slope** | **P-value** | **McFadden's pseudo-r2** |
| Female | Ventral | NB |  | 1356 | 0.516  (<10^-4^) | -0.291  (<10^-4^) | 0.005  (<10^-4^) | -3.978  (0.026) |  | 0.516 | -0.291 | 0.11 | 0.001 |
|  |  | BR |  | 1360 | 0.559  (<10^-4^) | -0.365  (<10^-4^) | <0.001  (<10^-4^) | -3.977  (0.026) |  | 0.559 | -0.365 | 0.046 | 0.002 |
|  | Dorsal | NB |  | 1338 | 1.399  (<10^-4^) | -1.057  (<10^-4^) | <0.001  (<10^-4^) | -3.983  (0.019) |  | 1.399 | -1.057 | <0.001 | 0.018 |
|  |  | BR |  | 1342 | 1.409  (<10^-4^) | -1.081  (<10^-4^) | <0.001  (<10^-4^) | -3.985  (0.020) |  | 1.409 | -1.081 | <0.001 | 0.019 |
| Male | Ventral | NB |  | 1105 | 0.521  (<10^-4^) | -0.400  (<10^-4^) | 0.001  (<10^-4^) | -3.973  (0.030) |  | 0.521 | -0.4 | 0.046 | 0.003 |
|  |  | BR |  | 1089 | 0.522  (<10^-4^) | -0.449  (<10^-4^) | <0.001  (<10^-4^) | -3.969  (0.039) |  | 0.522 | -0.449 | 0.028 | 0.003 |
|  | Dorsal | NB |  | 1213 | 1.425  (<10^-4^) | -1.301  (<10^-4^) | <0.001  (<10^-4^) | -3.985  (0.016) |  | 1.425 | -1.301 | <0.001 | 0.028 |
|  |  | BR |  | 1192 | 1.546  (<10^-4^) | -1.488  (<10^-4^) | <0.001  (<10^-4^) | -3.988  (0.013) |  | 1.546 | -1.488 | <0.001 | 0.035 |
| Juvenile | Ventral | N/A |  | 1226 | 0.731  (<10^-4^) | -0.185  (<10^-4^) | 0.071  (<10^-4^) | -3.985  (0.003) |  | 0.731 | -0.185 | 0.347 | 0.001 |
|  | Dorsal | N/A |  | 1109 | 1.47  (<10^-4^) | -0.641  (<10^-4^) | <0.001  (<10^-4^) | -3.982  (0.003) |  | 1.47 | -0.641 | 0.005 | 0.006 |

**Eco-regions’ avian assemblages analysis**


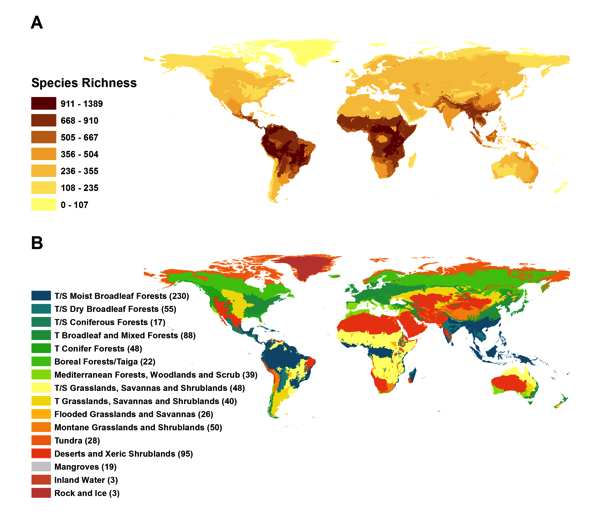


Fig. S1. Global distribution of avian species richness and habitat types in terrestrial eco-regions. A) The global distribution of avian species richness (breeding and non-breeding distributions). B) Each habitat type encompasses multiple eco-regions (numbers in brackets). Each type of habitat may be classified as either closed or open as indicated by colour: mostly closed = green and blue; mostly open = red, orange and yellow. Mangroves are indicated with grey, as they cannot be classified as either open or closed. T = Temperate; T/S = Tropical and subtropical. Eco-region spatial distributions were obtained from The Nature Conservancy.

Fig. S2. Correlations between the number of bird species with irregular vs. regular plumage patterns across avian assemblages in the world’s terrestrial eco-regions. This is shown for all the *biological combinations.*

Fig. S3. Correlations between the number of bird species with a plumage pattern versus without a plumage pattern across avian assemblages in the world’s terrestrial eco-regions. This is shown for all the *biological combinations.*

Table S3. Ratio of regular to irregular patterns and ratio of barred to mottled patterns from the total pool of avian species in the Western and Eastern hemispheres of the world for each *biological combination* (sex/breeding/age). Season: NB = Non-breeding; BR = Breeding.

| *Biological combination* | | | Total *ratio regular–irregular* | | Total *ratio barred–mottled* | |
| --- | --- | --- | --- | --- | --- | --- |
| **Sex** | **Body part** | **Season** | **Western hemisphere** | **Eastern hemisphere** | **Western hemisphere** | **Eastern hemisphere** |
| Female | Ventral | NB | 1.296 | 1.017 | 0.9 | 0.632 |
|  |  | BR | 1.288 | 1.02 | 0.888 | 0.637 |
|  | Dorsal | NB | 1.147 | 0.735 | 0.615 | 0.435 |
|  |  | BR | 1.141 | 0.74 | 0.607 | 0.442 |
| Male | Ventral | NB | 1.418 | 1.014 | 1.014 | 0.66 |
|  |  | BR | 1.465 | 1.064 | 1.019 | 0.686 |
|  | Dorsal | NB | 1.398 | 0.761 | 0.772 | 0.445 |
|  |  | BR | 1.365 | 0.766 | 0.74 | 0.444 |
| Juvenile | Ventral | N/A | 0.871 | 0.804 | 0.59 | 0.532 |
|  | Dorsal | N/A | 0.815 | 0.692 | 0.409 | 0.325 |

Fig. S4. Global spatial patterns of observed ratio *regular-irregular* across avian assembles in the world’s eco-regions. This is shown for all the *biological combinations.*

Table S4. Assemblage-level test of an association with habitat type for the ratio *barred-mottled*. For each *biological combination*, we present the total number of eco-regions in which we tested for an association, and the number, proportion and habitat type of eco-regions that have an observed ratio *barred-mottled* significantly different from the null expectation. Habitat type: TMBF = Tropical and Subtropical Moist Broadleaf Forests; TDBF = Tropical and Subtropical Dry Broadleaf Forests; MGS = Montane Grasslands and Shrublands; MFWS = Mediterranean Forests, Woodlands and Scrub; FGS = Flooded Grasslands and Savannas; DXS = Deserts and Xeric Shrublands. Season: NB = Non-breeding; BR = Breeding.

| **Biological combination** | | |  |  | **Significant eco-regions** | | |
| --- | --- | --- | --- | --- | --- | --- | --- |
| **Sex** | **Body part** | **Season** |  | **Number of eco-regions** | **Number** | **Proportion** | **Name** |
| Female | Ventral | NB |  | 766 | 1 | 0.001 | Antipodes Subantarctic Islands Tundra (Tundra – Australasia) |
|  |  | BR |  | 759 | 0 | 0 | – |
|  | Dorsal | NB |  | 769 | 0 | 0 | – |
|  |  | BR |  | 759 | 1 | 0.001 | Tibesti-Jebel Uweinat Montane Xeric Woodlands (DXS – Palearctic) |
| Male | Ventral | NB |  | 773 | 0 | 0 | – |
|  |  | BR |  | 767 | 0 | 0 | – |
|  | Dorsal | NB |  | 773 | 5 | 0.006 | Biak-Numfoor Rain Forests (TMBF – Australasia), Solomon Islands Rain Forests (TMBF – Australasia), Trobriand Islands Rain Forests (TMBF – Australasia), Kinabalu Montane Alpine Meadows (MGS – Indo-Malay), Murray-Darling Woodlands And Mallee (MFWS – Australasia) |
|  |  | BR |  | 767 | 3 | 0.004 | New Britain-New Ireland Lowland Rain Forests (TMBF – Australasia), Kinabalu Montane Alpine Meadows (MGS – Indo-Malay), Bohai Sea Saline Meadow (FGS – Palearctic) |
| Juvenile | Ventral | N/A |  | 763 | 1 | 0.001 | Antipodes Subantarctic Islands Tundra (Tundra – Australasia) |
|  | Dorsal | N/A |  | 761 | 0 | 0 | – |
